# The Degradation of TMEM166 by Autophagy Promotes AMPK Activation to Protect SH-SY5Y Cells Exposed to MPP^+^

**DOI:** 10.3390/cells11172706

**Published:** 2022-08-30

**Authors:** Zhaozhong Liao, Zunshuang Gong, Zhe Wang, Weiyan Yang, Wenjing Liu, Lin Hou, Xiaokun Liu, Junnan Hua, Bin Wang, Ning Li

**Affiliations:** 1 Department of Biochemistry and Molecular Biology, School of Basic Medicine, Qingdao University, Qingdao 266000, China; 2 Department of Biotechnology, School of Basic Medicine, Qingdao University, Qingdao 266000, China; 3 Department of Anesthesiology, Family Planning Service Center, Maternal and Child Health Hospital of Jiaozhou City, Qingdao 266000, China; 4 College of Electronic Information, Micro-Nano Technology College, Qingdao University, Qingdao 266000, China

**Keywords:** TMEM166, EVA1A, oxidative stress, Parkinson’s disease, mitochondria, AMPK, autophagy, mitophagy, PINK1/Parkin, SH-SY5Y cells

## Abstract

Neuronal oxidative stress caused by mitochondrial dysfunction plays a crucial role in the development of Parkinson’s disease (PD). Growing evidence shows that autophagy confers neuroprotection in oxidative-stress-associated PD. This work aims to investigate the involvement of TMEM166, an endoplasmic-reticulum-localized autophagy-regulating protein, in the process of PD-associated oxidative stress through the classic cellular PD model of neuroblastoma SH-SY5Y cells exposed to 1-methyl-4-phenylpyridinium (MPP^+^). Reactive oxygen species (ROS) production and mitochondrial membrane potential were checked to assess the oxidative stress induced by MPP^+^ and the cellular ATP generated was determined to evaluate mitochondrial function. The effect on autophagy induction was evaluated by analyzing p62 and LC3-II/I expression and by observing the LC3 puncta and the colocalization of LC3 with LAMP1/ LAMP2. The colocalization of mitochondria with LC3, the colocalization of Tom20 with LAMP1 and Tom20 expression were analyzed to evaluate mitophagy. We found that TMEM166 is up-regulated in transcript levels, but up-regulated first and then down-regulated by autophagic degradation in protein levels upon MPP^+^-treatment. Overexpression of TMEM166 induces mitochondria fragmentation and dysfunction and exacerbates MPP^+^-induced oxidative stress and cell viability reduction. Overexpression of TMEM166 is sufficient to induce autophagy and mitophagy and promotes autophagy and mitophagy under MPP^+^ treatment, while knockdown of TMEM166 inhibits basal autophagic degradation. In addition, overexpressed TMEM166 suppresses AMPK activation, while TMEM166 knockdown enhances AMPK activation. Pharmacological activation of AMPK alleviates the exacerbation of oxidative stress induced by TMEM166 overexpression and increases cell viability, while pharmacological inhibition mitophagy aggravates the oxidative stress induced by MPP^+^ treatment combined with TMEM166 overexpression. Finally, we find that overexpressed TMEM166 partially localizes to mitochondria and, simultaneously, the active AMPK in mitochondria is decreased. Collectively, these findings suggest that TMEM166 can translocate from ER to mitochondria and inhibit AMPK activation and, in response to mitochondrial oxidative stress, neuronal cells choose to up-regulate TMEM166 to promote autophagy/mitophagy; then, the enhancing autophagy/mitophagy degrades the TMEM166 to activate AMPK, by the two means to maintain cell survival. The continuous synthesis and degradation of TMEM166 in autophagy/mitochondria flux suggest that TMEM166 may act as an autophagy/mitochondria adaptor.

## 1. Introduction

Parkinson’s disease (PD) is a widespread neurodegenerative disease characterized by progressive loss of dopaminergic neurons in the substantia nigra pars compacta, part of the midbrain. According to current research, its etiology includes oxidative stress induced by mitochondrial dysfunction, neuroinflammation, abnormal protein folding, endoplasmic reticulum stress, the influence of the microbiota–gut–brain axis, genetic mutations and other environmental factors [1]; among them, oxidative stress is closely related to the development of PD [2]. However, the regulatory mechanism of oxidative stress in neurons of PD is still not clear.

Over the past 30 years, autophagy has gradually been found to be associated with PD, as the role of its familial gene (*SNCA*, *PINK1*, *Parkin*, *LRRK2* and *DJ-1*) mutations leads to dysfunction of autophagy, especially mitophagy [3,4,5]. Recent studies have shown that excessive oxidative stress and mitochondrial dysfunction are essential factors contributing to the initiation and development of PD [6,7,8]. It was found that PD animal models exhibit strongly dysfunctional autophagy, leading to α-syn aggregation and damaged mitochondria aggregation, while stimulating autophagy in genetic or pharmacological manners has shown protective effects in these models [9,10]. Therefore, methods to upregulate autophagy seem promising as strategies for PD treatment research.

TMEM166 (Transmembrane protein 166, also known as Eva-1 homologous protein A) was initially identified in a human kidney cDNA library in 2007 as an endoplasmic reticulum transmembrane protein related to apoptosis and autophagy [11]. Mechanistically, TMEM166 interacts with ATG16L1 (autophagy-related protein 16-like 1) to promote the formation of autophagic vesicles, thus, enhancing autophagy [12,13]. TMEM166 is further studied in cancer as a tumor suppressor gene [14,15,16,17]. Quantitative proteomics of TMEM166^−/−^ and wild-type mouse brains show that the differential proteins are related to ATP synthesis, oxidative phosphorylation and the tricarboxylic acid (TCA) cycle [18]. Further, combined with bioinformatics analysis and gene set enrichment analysis, these dysregulated proteins are associated with some neurodegenerative diseases, such as PD, Alzheimer’s disease and Huntington’s disease [18]. However, whether TMEM166 is related to PD and whether TMEM166-promoted autophagy confers protective effects on PD are unknown. In addition, a study found that liver-specific knockout of TMEM166 leads to mitochondrial damage in a mouse model of acute liver failure [19] and cardiomyocytes from TMEM166-knockout mice exhibit mitochondrial dislocation aggregation, leading to ATP deficiency [20]. More notably, mitochondria–endoplasmic reticulum crosstalk has proved that mitochondria form close physical contacts with a specialized domain of the ER, known as the mitochondria-associated membrane. These findings suggest a link between TMEM166 and mitochondrial function. Based on these studies, we chose SH-SY5Y neuroblastoma cells treated with MPP^+^ (1-methyl-4-phenylpyridinium) [21], a mitochondrial respiratory chain complex I inhibitor, as a neurotoxin cell model of PD, to initially investigate the function of TMEM166 in PD-associated oxidative stress and to explore its relationship with mitochondrial function.

AMPK (Adenosine 5′-monophosphate (AMP)-activated protein kinase) is an intracellular energy monitoring protein that has been considered a “guardian” of mitochondrial hemostasis for its various functions in mitochondria, such as biogenesis, fission, fusion and mitophagy [22]. Moreover, AMPK is an upstream regulator of autophagy and its activation inhibits MPP^+^-induced oxidative stress and confers neuroprotection in SH-SY5Y cells [23,24,25,26]. Thus, the regulation of AMPK in PD intrigues the interest of researchers.

Here, we first found that the degradation of TMEM166 by autophagy is essential for the survival of SH-SY5Y cells treated with MPP^+^ as a cell model of PD. Overexpression of TMEM166 attenuates the activation of AMPK in mitochondria and causes cells to be more vulnerable to MPP^+^. Therefore, cells instinctively degrade TMEM166 through autophagy to activate AMPK in the presence of MPP^+^, thereby promoting cell survival. This research indicates the potential of TMEM166 as a promising target to intervene in PD progression.

## 2. Materials and Methods

### 2.1. Materials

The Myc-TMEM166, LC3-mCherry and TMEM166-RFP plasmids were kindly provided by Prof. Wei Liu (Zhejiang university, Hangzhou, China). MPP^+^ (#36913-39-0) was obtained from Sigma-Aldrich (Shanghai, China). Cell Counting Kit (CCK-8, #CA1210) was from Solarbio Science & Technology (Beijing, China). ROS Assay Kit (#S0033S) and ATP Assay Kit (#S0026) were from Beytime Biotech (Shanghai, China). JC-1 Mitochondrial Membrane Potential Assay Kit and CCCP (#40706ES60) were from Yeasen Biotech (Shanghai, China). MG132 (#S2619) was from Selleck (Shanghai, China) and chloroquine (CQ #HY-17589A) was from MedChemExpress (Shanghai, China). Cyclosporin A (CsA #ab120114) was from Abcam (Shanghai, China). AICAR (#HY-13417) was from MedChemExpress (Shanghai, China). TMEM166 (#ab216043) antibody was from Abcam. P62 (#18420-1-AP) antibody, Tom20 (#11802-1-AP) antibody, VDAC (#10866-1-AP) antibody, PINK1 (#23274-1-AP) antibody, total-AMPK (#66536-1-Ig) antibody, LAMP1 (#65051-1-lg) antibody and LAMP2 (#27823-1-AP) antibody were from Proteintech (Wuhan, China). LC3 (#M186-3) antibody was from MBL (Beijing, China). β-actin (#GB11001) antibody was from Servicebio (Wuhan, China). GAPDH (#AC001) antibody was from Abclonal (Wuhan, China). Parkin (#2132) antibody and phospho-AMPK (#2535) antibody were from Cell Signaling Technology (Shanghai, China). FITC Goat anti-Rabbit IgG (#AS011), Alexa Fluor 488-conjugated Goat anti-Mouse IgG (#AS037) and Alexa Fluor 594-conjugated Goat anti-Mouse IgG (#AS054) secondary antibody were from Abclonal (Wuhan, China).

### 2.2. Cell Culture and Transfection

The neuroblastoma cell line SH-SY5Y was obtained from National Collection of Authenticated Cell Cultures and grown in DMEM (#L110KJ, Basalmedia, Shanghai, China) with 10% FBS (#CCS30009.02 500 mL, EN MOASAI BIOLOGICAL TECHNOLOGY CO., LTD, Changzhou, China ) at cell incubator. Plasmid DNA transfection was performed according to the manufacturer’s guidelines of Lipofectamine 2000 (#11668019, Invitrogen, Carlsbad, CA, USA).

### 2.3. TMEM166 Knock Down

Specific double-stranded siRNA against TMEM166 (si-TMEM166: sense 5′-UGAUAAGGAUCUCUUGCCATT-3′; antisense 5′-UGGCAAGAGAUCCUUAUCATT-3′) was synthesized by GenePharma Corporation (Shanghai, China). A scrambled siRNA that has no sequence homology to any known human genes was regarded as the negative control (si-n.c.). The siRNA transfection was performed according to the manufacturer’s guidelines of Lipofectamine 2000 reagent (Invitrogen, Carlsbad, CA, USA), knocking down twice for a total of 72 h.

### 2.4. RNA Isolation and Real-Time Polymerase Chain Reaction (RT-PCR)

Total RNA was extracted from cells using Trizol (TaKaRa Biomedical Technology (Beijing) Co., Ltd., Beijing, China) according to the manufacturer’s instructions. Complementary DNA was synthesized using the Cdna Reverse Transcription Kit (HiScript^®^ II Q RT superMix, Vazyme, Nanjing, China), the expression of Mrna was analyzed by quantitative real-time PCR with SYBR Green Master Mix (ChamQTM SYBR^®^ Color Qpcr Master Mix, Vazyme, Nanjing, China). Real-time PCR was performed with a CFX96 Touch Real-Time PCR Detection System (Bio-Rad, Hercules, CA, USA). mRNA levels were analyzed by quantitative RT-PCR (qRT-PCR) and normalized to the levels of the Gapdh housekeeping. qRT-PCR assays were performed in triplicate for each sample. The primers used for qRT-qPCR are listed below.

The used primers were:

TMEM166-forward, 5′-AGATGGCTTTGCTCAGCAACA-3′

TMEM166-reverse, 5′-GATGCACACGCCAGAAACAA-3′

GAPDH-forward, 5′-AACGGATTTGGTCGTATTGGG-3′

GAPDH-reverse, 5′-TCGCTCCTGGAAGATGGTGAT-3′

### 2.5. Mitochondrial Fraction Isolation

The extraction method is slightly modified from Mariusz R Wieckowski et al. [27]. All the reagents were freshly prepared and the final mitochondria fraction was lysed in RIPA lysis buffer with 1% (*v*/*v*) PMSF and phosphatase inhibitor.

### 2.6. Western Blotting Analysis

Cells were lysed in RIPA lysis buffer with 1% (*v*/*v*) PMSF and phosphatase inhibitor to obtain protein samples, then using the BCA protein quantification kit (Solarbio, Beijing, China) to estimate the protein concentrations. All samples were boiled in 1 × SDS sample buffer and separated by SDS-PAGE and subsequently transferred onto PVDF membranes (Millipore). After blocking with 5% skimmed milk or BSA in TBST, the membranes were incubated with the primary antibodies overnight at 4 °C. Then the blot was incubated with peroxidase-conjugated sheep anti-rabbit IgG (Servicebio, Beijing, China) or HRP Conjugated AffiniPure goat-anti-mouse IgG (H + L) (BOSTER, Wuhan, China). ECL system was used to capture blot images. Each independent experiment was performed at least three times.

### 2.7. Cell Viability Assay

The relative cell viability was measured by Cell Counting Kit (CCK-8) assay. Cells to be tested were seeds in a 96-well plate containing 2000 cells each group in cell incubator. After incubating for the indicated times, culturing plate was replaced with 110 µL medium containing 10 µL CCK8 reagent per well and incubated for 4 h. The absorbance of the samples was measured at 450 nm by plate reader (Bio Tek, Beijing, China). Each independent experiment was performed at least three times.

### 2.8. Annexin V/PI Apoptosis Analysis and PI Cell Cycle Analysis

Annexin V/PI Apoptosis Detection Kit (Solarbio, Beijing, China) was used to evaluate cell Apoptosis rate according to the manufacturer’s instructions. Briefly, cells to be tested were harvested using trypsin without EDTA, washed twice with cold PBS and once with cold binding buffer, then resuspended with cold binding buffer and incubated with Annexin V-FITC and PI staining solution for 15 min at room temperature in the dark. Samples were analyzed by flow cytometer (Becton Dickinson, Franklin Lakes, NJ, USA). Each independent experiment was performed at least three times.

PI staining was used to detect cell cycle. In brief, cells to be tested were collected and washed with cold PBS three times and resuspended with 70% cold ethanol at 4 °C overnight. Then, cells were stained with 50 µg/mL PI containing RNase (100 µg/mL) for 30 min. The results were obtained from cytometry. Each experiment was performed at least three times.

### 2.9. Mitochondrial Membrane Potential Analysis

JC-1 assay kit (Yeasen, Shanghai, China) was used to determine the mitochondrial membrane potential according to the manufacturer’s instructions. The JC-1 dye aggregates in the healthy mitochondria and emits red fluorescence. However, in unhealthy cells, due to the drop or loss of mitochondrial membrane potential, the JC-1 dye cannot aggregate in the mitochondria, remaining as monomers in the cytoplasm and emitting green fluorescence. Cells to be detected were washed with PBS and stained with JC-1 reagent for 20 min and then washed with culture medium three times. The fluorescence was observed under a fluorescence microscope (Nikon, Shanghai, China).

### 2.10. Reactive Oxygen Species (ROS) Analysis

Intracellular ROS levels were determined by the non-fluorescent probe 2, 7-dichlorofluorescein diacetate (DCFH-DA) (Beyotime Biotechnology, Shanghai, China) according to the manufacturer’s instructions. Briefly, cells to be detected were washed twice with culture medium, then incubated with 10 µM DCFH-DA probe of final concentration for 20 min in the cell incubator. The DCF green fluorescence was observed by fluorescence microscope at excitation wavelength 488 nm.

### 2.11. Measurement of ATP Levels

Intracellular ATP levels were determined by the ATP Assay Kit (Beyotime Biotechnology, Shanghai, China) according to the manufacturer’s instructions. In brief, ATP detection working buffer (100 µL) was gently mixed with the substrate, then luminescence was measured using a micro-plate reader (Bio Tek, Beijing, China). Each independent experiment was performed at least three times.

### 2.12. Immunocytochemical Staining and Confocal Microscopy

For immunostaining, cells seeded on cover slips were fixed in 4% formaldehyde for 15 min, washed twice with PBS and permeabilized with 0.2% Triton X-100 (Beyotime Biotechnology, Shanghai, China) for 20 min. Then cells were incubated in blocking buffer (Normal Goat Serum, BOSTER, Wuhan, China) for 60 min. Cells were then incubated with appropriate primary antibodies and secondary antibodies for 1 h separately. Cells transfected with LC3-Cherry or TMEM166-RFP were stained with MitoTracker according to the manufacturer and observed under a laser scanning confocal microscope (STELLARIS 5, Leica) with a 63 × Plan Apochromat 1.4 NA objective. Live cell imaging was performed in LabTek chambers (Nalge Nunc International) maintained at 37 °C with 5% CO_2_. For quantification of the number of LC3 dots and colocalization dots per cell, a total of 20 images was recorded and analyzed.

### 2.13. Statistical Analysis

All experiments were conducted at least three independent times. Data are ex-pressed as the mean ±standard deviation (SD). GraphPad Prism 6 software (San Diego, California, CA, USA) was used for all statistical analyses; the differences between two independent groups were analyzed using two-tailed Student’s t test and differences were considered statistically significant at *p <* 0.05.

## 3. Results

### 3.1. TMEM166 Is First Up-Regulated and Then Down-Regulated by Autophagic Degradation in MPP^+^-Treated SH-SY5Y Cells

**Figure 1:**
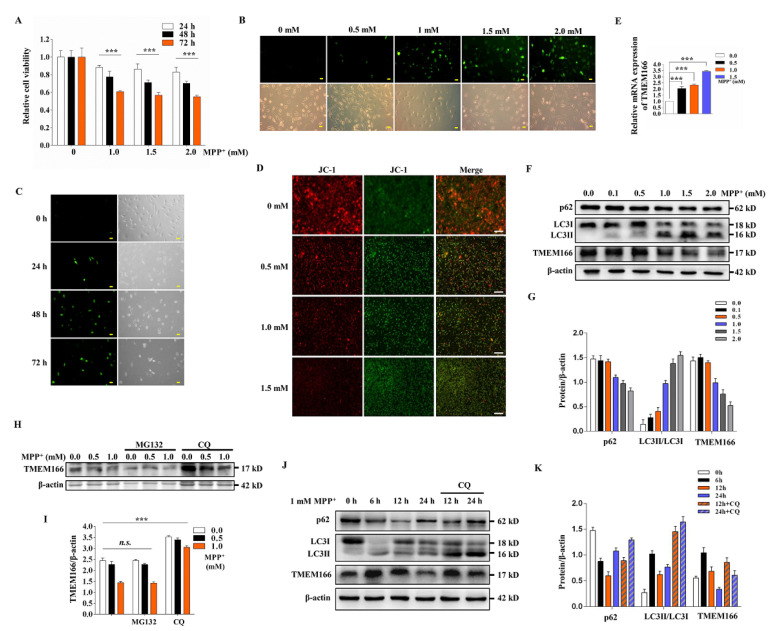
TMEM166 is degraded by autophagy under MPP^+^-induced oxidative stress. (**A**) Relative viability of SH-SY5Y cells after treatment with different concentrations of MPP^+^ for the indicated times. *** *p* < 0.001 vs. 0 mM (n = 4; means ± SDs). (**B**) SH-SY5Y cells were treated with MPP^+^ at the indicated concentrations for 24 h and then intracellular reactive oxygen species (ROS) were detected by DCFH-DA staining and visualized by fluorescence microscopy. Scale bars, 50 μm. (**C**) SH-SY5Y cells were treated with 1 mM MPP^+^ for the indicated time and then intracellular ROS were detected. Scale bars, 50 μm. (**D**) SH-SY5Y cells were treated with MPP^+^ at the indicated concentrations for 24 h and then the mitochondrial membrane potential was detected by JC-1 staining and visualized by fluorescence microscopy. Scale bars, 500 μm. (**E**,**F**) SH-SY5Y cells exposed to MPP^+^ at the indicated concentrations for 24 h were assessed for TMEM166 mRNA levels by qRT–PCR and its protein levels by Western blot. Relative mRNA expression levels were normalized to the 0.0 mM control. *** *p* < 0.001 vs. 0.0 mM (n = 3; means ± SDs). β-actin was used as a loading control. (**G**) Qualification analysis of blot bands of protein from (**F**) with ImageJ v1.53k. (**H**) SH-SY5Y cells were exposed to MPP^+^ at the indicated concentrations for 24 h with or without MG132 (3 μM) or CQ (20 μM) and TMEM166 levels were determined by Western blot. (**I**) Qualification analysis of blot bands of TMEM166 from (**H**). *** *p* < 0.001, n.s. *p* ≥ 0.05 (n = 3; means ± SDs). (**J**) SH-SY5Y cells were treated with 1 mM MPP^+^ for the indicated time in the presence or absence of CQ (50 μM, last 1.5 h) and protein levels were determined by Western blot. (**K**) Qualification analysis of blot bands of protein from (**J**).

Using the classic PD cell model of human dopaminergic SH-SY5Y cells treated with the mitochondrial respiratory chain blocker MPP^+^, we explored the effect of MPP^+^ on cell viability. The CCK-8 assay results showed that with increasing MPP^+^ concentration and treatment time, cell viability gradually decreased (Figure 1A). Reactive oxygen species (ROS) analysis demonstrated that the longer the MPP^+^ treatment time was, the more ROS were produced (Figure 1C) and the higher the treatment concentration was, the more ROS were produced (Figure 1B) and the more severe decline in mitochondrial membrane potential (MMP) (Figure 1D). The cell morphology after 72 h of treatment was significantly abnormal, indicating that MPP^+^ treatment causes SH-SY5Y cell death (Figure 1C). Meanwhile, we detected the changes in TMEM166 mRNA and protein levels in SH-SY5Y cells after MPP^+^ treatment by qRT-PCR and Western blot. It was found that with the increase in the MPP^+^ treatment concentration, the mRNA level of TMEM166 significantly increased (*p* < 0.001, Figure 1E). However, its protein level gradually decreased, especially after 1.0 mM MPP^+^ treatment, while the autophagic membrane protein LC3II/LC3I levels gradually increased and the autophagy adaptor protein p62 levels gradually decreased (Figure 1F,G), suggesting that with the MPP^+^ treatment, autophagy is up-regulated and TMEM166 protein is degraded. To determine how TMEM166 degrades, we compared and evaluated the degradation of TMEM166 under MPP^+^ treatment combined with the proteasome inhibitor MG132 or the lysosome inhibitor chloroquine (CQ). CQ treatment significantly inhibited the decline in TMEM166 protein levels caused by MPP^+^ treatment (*p* < 0.001, Figure 1H,I), while MG132 treatment did not prevent the degradation of TMEM166 protein (Figure 1H,I), indicating that TMEM166 is not degraded by ubiquitin–proteasome but by autophagy.

Although the protein levels of TMEM166 started to drop after 24 h of MPP^+^ treatment (Figure 1F,G), the transcript level was elevated (*p* < 0.001, Figure 1E), implying TMEM166 may not be degraded at first. In fact, the expression levels of TMEM166 increased at 6 h after 1 mM MPP^+^ treatment and then decreased gradually; accompanied by this, the degradation of p62 and the conversion of LC3I to LC3II also increased first but then only slightly decreased and CQ treatment inhibited the degradation of TMEM166. Meanwhile, it also inhibited the degradation of p62 and the conversion of LC3 II to LC3 I (Figure 1J,K). This result indicated that at the beginning of MPP^+^ treatment, TMEM166 is synthesized faster than it is degraded, so its protein levels rise, possibly leading to up-regulation of autophagy. In turn, up-regulated autophagy (not excluding TMEM166 independent) accelerates the degradation of TMEM166 beyond its synthesis rate, so that TMEM166 protein levels begin to decline and gradually fall below basal levels, while autophagy remained at a high level after its rise.

Taken together, these data suggest TMEM166 is up-regulated and then down-regulated by autophagic degradation in MPP^+^-induced oxidative stress. TMEM166 could be an autophagic adaptor protein.

### 3.2. Overexpressed TMEM166 Exacerbates MPP^+^-Induced Oxidative Stress in SH-SY5Y Cells

**Figure 2:**
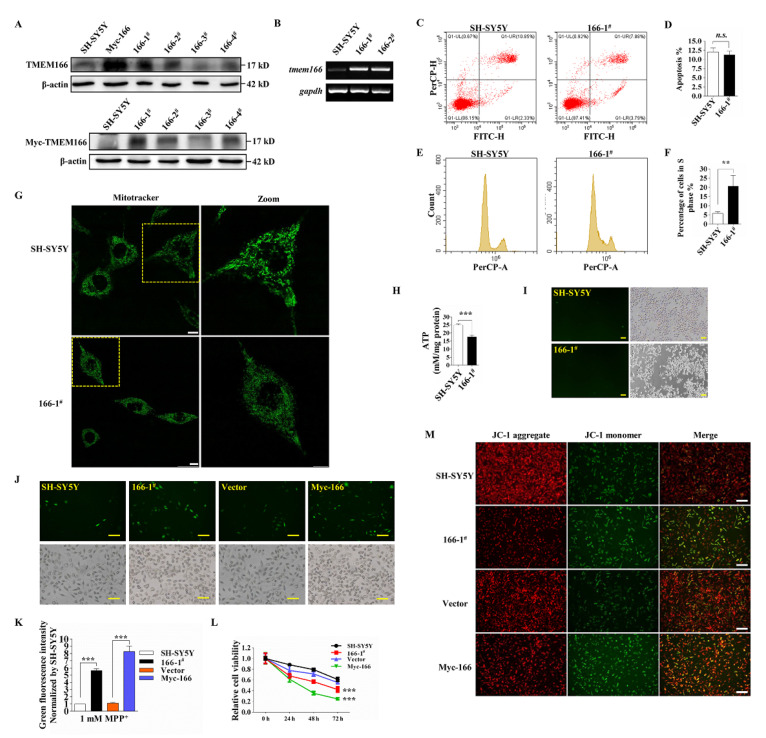
Overexpression of TMEM166 exacerbates MPP^+^-induced oxidative stress. (**A**) SH-SY5Y cells were transfected with Myc-TMEM166 plasmid, then the stable expression cell clones were obtained by G418 screening and the expression of TMEM166 or Myc-TMEM166 was detected by Western blot using TMEM166 antibody or Myc-tag antibody. (**B**) Agarose gel electrophoresis was performed to determine the tmem166 cDNA of stable expression cell clones “166-1^#^” and “166-2^#^”. Gapdh was used as a loading control. (**C**) Flow cytometry for apoptosis of cells with stable expression of TMEM166 through Annexin V-FITC/PI staining. (**D**) Quantification of apoptosis rates from (**C**). n.s. *p* ≥ 0.05 (n = 4; means ± SDs). (**E**) Flow cytometry for the cell cycle of cells with stable TMEM166 expression through PI staining. (**F**) Quantification of the percentage of cells in each cell cycle stage. ** *p* < 0.01 (n = 4; means ± SDs). (**G**) The morphology of mitochondria was visualized by confocal microscope confocal after MitoTracker green staining. Scale bars, 10 μm. (**H**) Mitochondrial ATP content was measured in SH-SY5Y and 166-1^#^ cells. (**I**) ROS were detected in SH-SY5Y and 166-1^#^ cells. Scale bars, 50 μm. (**J**) SH-SY5Y, 166-1^#^ or Myc-166 (SH-SY5Y cells transiently transfected with Myc-TMEM166) cells were exposed to 1 mM MPP^+^ for 24 h, followed by ROS detection. Scale bars, 50 μm. (**K**) Quantification analysis of the ROS fluorescence intensity with ImageJ v1.53k. Data were normalized to the SH-SY5Y control. *** *p* < 0.001 (n = 4; means ± SDs). (**L**) Relative cell activity detection by CCK8 assay after 1 mM MPP^+^ treatment at indicated time. *** *p* < 0.001 (n = 4; means ± SDs). (**M**) The mitochondrial membrane potential was evaluated by JC-1 staining after 1 mM MPP^+^ treatment for 24 h. Scale bars, 100 μm.

Numerous studies have shown that TMEM166 is up-regulated and involved in tumor suppression, chemotherapeutic resistance and acute liver failure by promoting autophagy and/or apoptosis [11,13,14,15,16,19], while in the present study, TMEM166 was first up-regulated and then down-regulated in MPP^+^-induced oxidative stress (Figure 1J). The down-regulation hints that TMEM166 may perform other functions in this situation. To explore the function of TMEM166 during MPP^+^-induced oxidative stress, SH-SY5Y cells were transfected with Myc-TMEM166 plasmid, subjected to resistance screening to construct stably expressing TMEM166 cell lines. The “166-1^#^” and “166-2^#^” cell lines were obtained. We first performed Western blot and agarose gel electrophoresis assays to confirm the stable overexpression of TMEM166 (Figure 2A,B). Using flow cytometry to determine the apoptosis and cell cycle of cells, we found that the apoptosis rates of the TMEM166 overexpression group “166-1^#^” showed little change compared with those of the SH-SY5Y group (*p* ≥ 0.05, Figure 2C,D), but the cell cycle of the 166-1^#^ cells clearly arrested in the S phase (*p* < 0.01, Figure 2E,F). Then, we detected the mitochondrial morphology and ATP production capacity of 166-1^#^ cells. Surprisingly, the mitochondria of 166-1^#^ cells were swollen, appeared more granular, short rod shaped and diffused, while those of the SH-SY5Y group were linear and network-like stretching (Figure 2G). The ATP production capacity of 166-1^#^ cells was significantly lower than that of SH-SY5Y cells (*p* < 0.001, Figure 2H), but 166-1^#^ cells did not produce ROS per se (Figure 2I), indicating that the energy-producing function of mitochondria was impaired by overexpressed TMEM166 and TMEM166 itself does not cause oxidative stress.

Next, we assessed the effect of MPP^+^-induced oxidative stress on TMEM166 overexpressing cells. Since there is no large loss of dopaminergic neurons in the early stage of PD patients, 1 mM MPP^+^ for 24 h was used to simulate the process of oxidative stress in the PD cell model for further experiments. We found that overexpression of TMEM166 increased MPP^+^-induced ROS production (*p* < 0.001, Figure 2J,K) and decreased cell viability, regardless of transient transfection or stable expression (*p* < 0.001, Figure 2L), and the results of JC-1 staining of MMP showed that the healthy MMP (red) of the 166-1^#^ and Myc-166 groups decreased significantly and represented unhealthy MMP (green) increased significantly compared with SH-SY5Y groups (Figure 2M). Together, these data suggest that overexpression of TMEM166 aggravated MPP^+^-induced oxidative stress and MMP decline.

### 3.3. TMEM166 Promotes Autophagy in SH-SY5Y Cells

**Figure 3:**
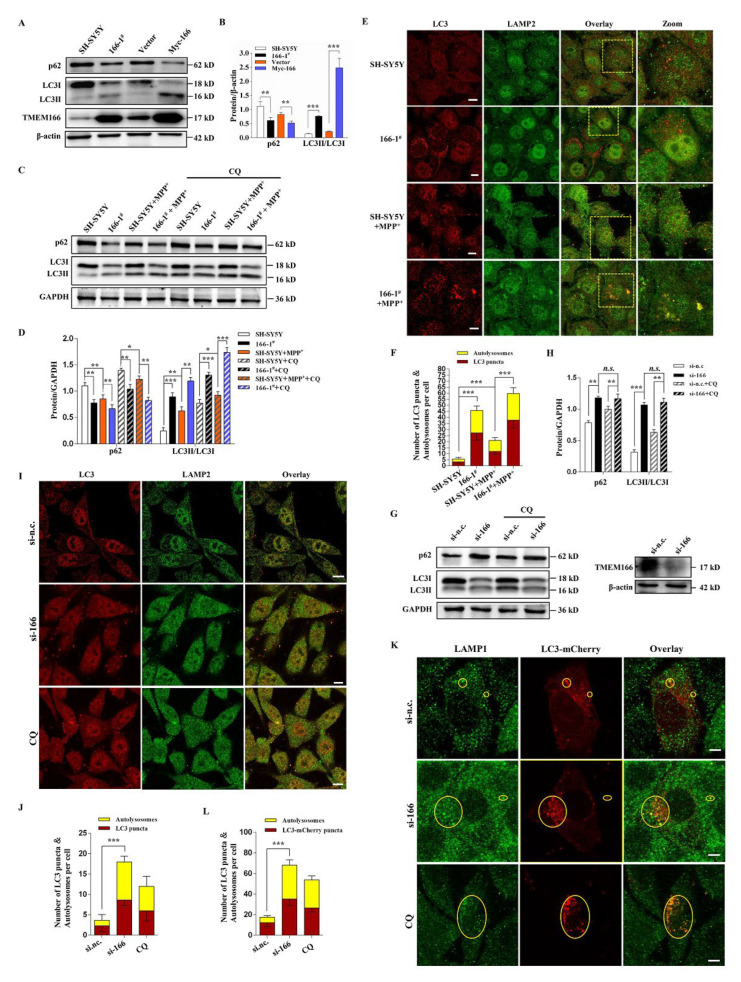
Overexpressed TMEM166 promotes autophagy and knockdown TMEM166 inhibits autophagic degradation. (**A**) SH-SY5Y cells or 166-1^#^ or overexpressed Myc-166 cells were lysed and the autophagy-related protein levels were determined. (**B**) Quantification analysis of protein levels from (**A**). (**C**) SH-SY5Y cells and 166-1^#^ cells were untreated or treated with 1 mM MPP^+^ for 24 h in the presence or absence of 50 µM CQ (last 1.5 h) and the expression levels of autophagy-related proteins were detected by Western blot. (**D**) Quantification analysis of protein levels from (**C**). (**E**) Untreated or treated with 1 mM MPP^+^ for 24 h, SH-SY5Y cells and 166-1^#^ cells were fixed and stained with LC3 and LAMP2 antibodies and were imaged by confocal microscopy. Scale bars, 10 µm. (**F**) Statistical analysis of the number of LC3 puncta and the colocalized dots of LC3 with LAMP2 (autolysosomes) per cell from (**E**). (n = 20; means ± SDs) (**G**) SH-SY5Y cells were transfected with TMEM166 siRNA (si-166) or si-n.c. (nonspecific control) for 72 h with or without CQ (50 µM, last 1.5 h), then the interference effect was verified and autophagy-related proteins were detected by Western bot. (**H**) Quantification analysis of protein levels from (**G**). (**I**) SH-SY5Y cells transfected with si-166 or si-n.c. or treated with 50µM CQ for 1.5 h were fixed and stained with LC3 and LAMP2 antibodies and were imaged by confocal microscopy. Scale bars, 10 µm. (**J**) Statistical analysis of the number of LC3-dots and the colocalized dots of LC3 with LAMP2 (autolysosomes) per cell from (**I**). (**K**) SH-SY5Y cells were transfected with LC3-mCherry plasmids, then were treated as indicated, fixed and stained with LC3 and LAMP1 antibodies and were imaged by confocal microscopy. Scale bars, 5 µm. (**L**) Statistical analysis of the number of LC3-mCherry dots and the colocalized dots of LC3-mCherry with LAMP1 per cell from (**K**). Scale bars, 5 µm. * *p* < 0.05, ** *p* < 0.01, *** *p* < 0.001.

According to the previous studies, TMEM166 is an important autophagy regulator [11,12], so we inferred that during the process of MPP^+^-induced oxidative stress, more and more damaged mitochondria were produced and cells instinctively up-regulate TMEM166 to promote autophagy to degrade the damaged mitochondria to maintain cell homeostasis. Therefore, we compared the autophagy level in the two TMEM166 overexpression groups (166-1^#^ and Myc-166) and the SH-SY5Y group and the results showed that overexpressed TMEM166 induced a significant enhancement in the conversion of LC3-I to LC3-II, a significant decrease in p62 (an indicator of autophagic degradation) levels (Figure 3A–D) and a dramatic increase in number of endogenous LC3 punta (autophagosome marker) and the colocalized dots of LC3 with LAMP2 (lysosomal-associated membrane protein 2, a marker of lysosome) (*p* < 0.001, Figure 3E,F). The colocalized dots are called autolysosomes, which reflect the amount of cargo to be degraded by autophagy. The number of autophagosomes and autophagosome–lysosome fusion were further separately studied by observing the LC3-mCherry puncta and the colocalization of LC3-mCherry with endogenous LAMP1 (lysosomal-associated membrane protein 1). As shown in Supplementary Figure S1, overexpression of TMEM166 caused a significant growth in numbers of LC3-mCherry puncta and a significant increase in colocalization of LC3-mCherry with LAMP1. In addition, CQ treatment led to a further increase in LC3II/LC3I levels and p62 levels both in 166-1^#^ and SH-SY5Y group (Figure 3C,D). Upon MPP^+^-treatment, autophagosome formation and autophagic degradation were still stronger in 166-1^#^ cells than in SH-SY5Y cells (Figure 3C–F and Figure S1). These data suggest that overexpressed TMEM166 promotes autophagic flux, which is consistent with previous studies [12]. Notably, MPP^+^ treatment also promotes autophagic flux, which was weaker than the TMEM166 overexpression initiated (Figure 3C–F and Figure S1).

Then, to further clarify the role of 166 in autophagy, we knocked down TMEM166 with siRNA in SH-SY5Y cells and checked the changes on the basal-level autophagy. Compared with si control group, knockdown of TMEM166 induced a dramatic increase in the conversion of LC3-I to LC3-II (*p* < 0.001, Figure 3G,H) and a significant accumulation of p62 protein (*p* < 0.01, Figure 3G,H) and CQ treatment promoted the accumulation of p62 and LC3 II in control cells but not in TMEM166 knockdown cells (Figure 3G,H), indicating that basal autophagic degradation was blocked when TMEM166 was knocked down. In search of further evidence that knockdown of TMEM166 inhibits basal autophagic degradation, we observed intracellular autophagosomes and autolysosomes. We found knockdown of TMEM166 increased LC3 punta or LC3-mCherry punta and the colocalization of LC3 with LAMP2 or the colocalization of LC3-mCherry with LAMP1 (*p* < 0.001, Figure 3I–L), but the colocalization was almost 100% (Figure 3J,L), which means that almost all autophagosomes in TMEM166 knockdown cells are fused to lysosomes, mimicking the effect of CQ. Taken together, these data suggest TMEM166 knockdown does not affect the autophagosome–lysosome fusion but inhibits the autophagic degradation.

Collectively, these findings illustrate that increased expression of TMEM166 is sufficient to induce autophagy, and TMEM166 is required for basal autophagic degradation.

### 3.4. TMEM166 Promotes Mitophagy in SH-SY5Y Cells

**Figure 4:**
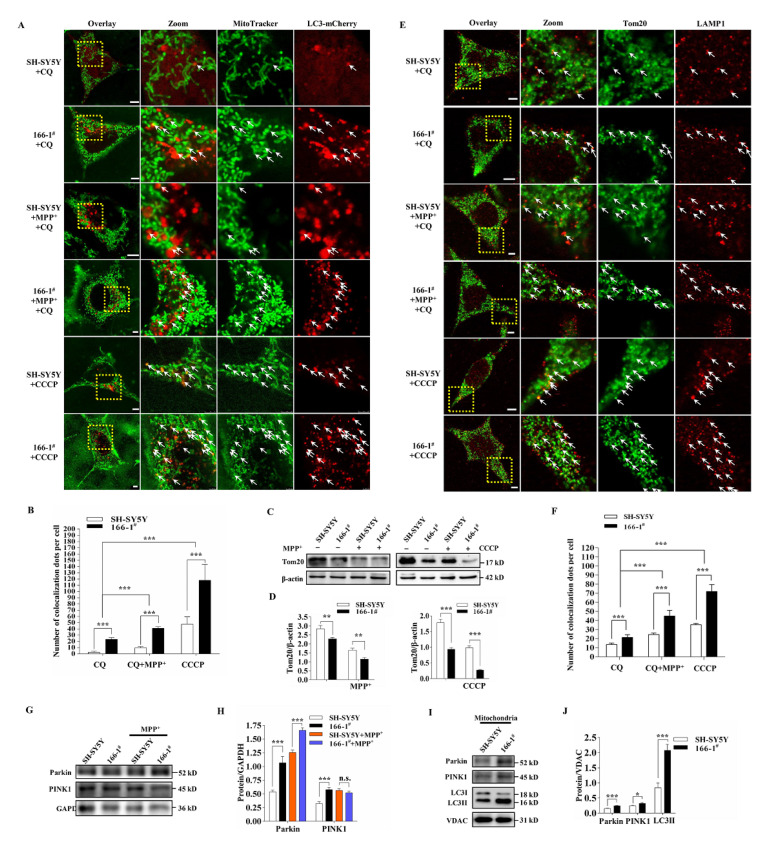
Overexpressed TMEM166 promotes mitophagy. (**A**) SH-SY5Y and 166-1^#^ cells transfected with LC3-mCherry were treated with 50 μM CQ for 1.5 h or 1 mM MPP^+^ for 24 h combined with CQ (50 μM, last 1.5 h) or 10 μM CCCP for 6 h, then were stained with MitoTracker and we performed the live cell imaging. Arrows present the colocalization dots of mitochondria and LC3. Scale bars, 5 μm. (**B**) Statistical data of colocalization dots of LC3-mCherry with mitochondria per cell are shown as the means ± SDs from 20 cells of duplicate experiments. (**C**)SH-SY5Y and 166-1^#^ cells were treated with 1 mM MPP^+^ for 24 h or 10 μM CCCP for 6 h and the expression level of the mitochondrial outer-membrane protein Tom20 was detected by Western blot. (**D**) Quantification analysis of Tom20 levels from (**C**). (**E**) SH-SY5Y and 166-1^#^ cells were treated as indicated, fixed and stained with Tom20 and LAMP1 antibodies and were imaged by confocal microscopy. Arrows present the colocalization dots of Tom20 and LAMP1. Scale bars, 5 µm. (**F**) Statistical data of colocalization dots of Tom20 with LAMP1 per cell are shown as the means ± SDs from 20 cells. (**G**,**H**) Parkin and PINK1 levels in SH-SY5Y cells and 166-1^#^ cells with or without MPP^+^ treatment. (**I**,**J**) Parkin, PINK1 and LC3 levels in the mitochondria of SH-SY5Y cells and 166-1^#^ cells. VDAC was used as a loading control. * *p <* 0.05, ** *p <* 0.01, *** *p <* 0.001.

Next, we explored whether TMEM166 also directly regulates mitophagy. We first observed the colocalization of mitochondria (MitoTracker staining, Green) and LC3-mCherry (Red), which represents the mitophagosome amounts. Live cell imaging results showed that, whether treated with MPP^+^ or not, compared with SH-SY5Y cells, 166-1^#^ cells showed more mitochondrial colocalization with LC3-mCherry when autophagic flux was blocked by CQ (*p* < 0.001, Figure 4A,B) and their colocalization was significantly increased in both SH-SY5Y and 166-1^#^ cells upon MPP^+^ treatment (*p* < 0.001, Figure 4A,B). When treated with CCCP, an uncoupler of mitochondrial oxidative phosphorylation and a classical mitophagy inducer, the colocalization of mitochondria with LC3 in 166-1^#^ cells was significantly more than that in SH-SY5Y cells (*p* < 0.001, Figure 4A,B). These results implied that TMEM166 could promote the production of mitophagosome or damaged mitochondria or facilitate mitochondria–autophagosome fusion. To further verify the promoting effect of TMEM166 on mitophagy, we determined the mitochondrial outer-membrane protein Tom20 level, whose decreased level reflects the mitophagy level. We found that compared with SH-SY5Y cells, the level of Tom20 in 166-1^#^ cells decreased significantly and a similar result was shown for the cells treated with MPP^+^ or CCCP (Figure 4C,D). In addition, more LC3II protein was detected in the isolated mitochondria of 166-1^#^ cells (*p* < 0.001, Figure 4I,J), indicating that cells overexpressing TMEM166 recruited more autophagosomes to mitochondria. We also examined the colocalization of Tom20 with LAMP1. It was demonstrated that upon 1.5 h CQ treatment, 166-1^#^ cells showed more colocalization of Tom20 with LAMP1 than SH-SY5Y cells (*p* < 0.001, Figure 4E,F) and their colocalization was significantly elevated after MPP^+^ or CCCP treatment (*p* < 0.001, Figure 4E,F), which is consistent with the results of colocalization of MitoTracker with LC3-mCherry. Combined with the decrease in Tom20 level and the increase in mitophagosome amounts, these data demonstrate that overexpressed TMEM166 can promote mitophagy.

Furthermore, we explored whether TMEM166-promoted mitophagy occurs through the canonical mitophagy pathway, PINK1/Parkin-dependent mitophagy. Western blot results showed that compared with SH-SY5Y cells, the levels of Parkin and PINK1 proteins in 166-1^#^ cells were significantly increased (*p* < 0.001, Figure 4G,H) and under the condition of MPP^+^ treatment, Parkin levels in 166-1^#^ cells were still obviously higher than those in SH-SY5Y cells, while the difference in PINK1 levels was not statistically significant (Figure 4G,H). In addition, we extracted mitochondrial proteins and found that the levels of PINK1 and Parkin in the mitochondria of 166-1^#^ cells were significantly higher than those in SH-SY5Y cells (Figure 4I,J), suggesting that TMEM166-promoted mitophagy should be PINK1/Parkin dependent.

### 3.5. Not Mitophagy but Inactivation of AMPK Induced by Up-Regulated TMEM166 Aggravates MPP^+^-Induced Oxidative Stress

**Figure 5:**
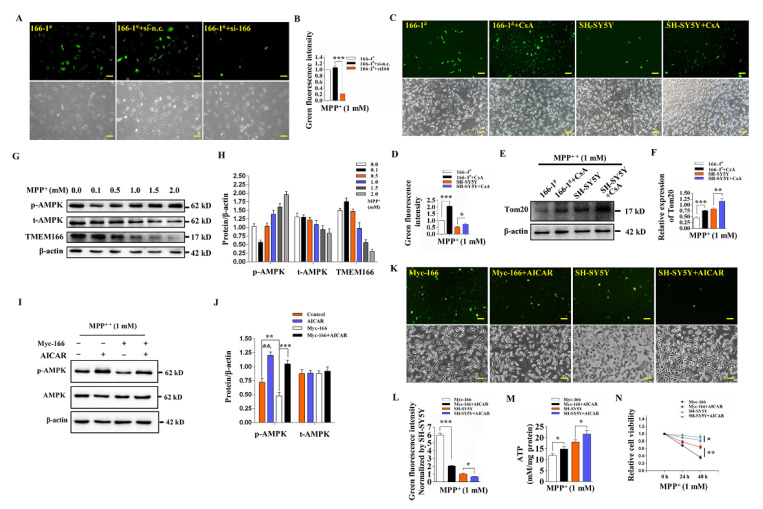
Overexpressed TMEM166 aggravates MPP^+^-induced oxidative stress by inhibiting AMPK activation independent of mitophagy promotion. (**A**) 166-1^#^ cells after knocking down TMEM166 were exposed to MPP^+^ for 24 h and then ROS production was detected. Scale bars, 50 μm. (**B**) Quantification analysis of the ROS fluorescence intensity with ImageJ v1.53 k. Data were normalized to the 166-1^#^ control. (**C**,**E**) 166-1^#^ cells and SH-SY5Y cells were pretreated with 0.4 μM CsA for 1 h and then co-treated with 1 mM MPP^+^ for 24 h. ROS production (**C**) and Tom20 protein levels (**E**) were detected. Scale bars, 50 μm. (**D**,**F**) Qualification analysis of the ROS fluorescence intensity from (**C**) and blot bands of Tom20 from (**E**). (**G**) SH-SY5Y cells were exposed to MPP^+^ at the indicated concentrations for 24 h and the p-AMPK, total AMPK and TMEM166 levels were determined by Western blot. (**H**) Quantification analysis of protein levels from (**G**). (**I**–**M**) SH-SY5Y cells or SH-SY5Y cells transfected with Myc-TMEM166 were treated with MPP^+^ in the presence or absence of AICAR (2 mM, last 5 h) and then the p-AMPK level (**I**,**J**), production of ROS (**K**,**L**) and ATP production capacity (**M**) were detected. (**N**) Relative cell activity of SH-SY5Y cells or SH-SY5Y cells transfected with Myc-TMEM166 exposed to 1 mM MPP^+^ for 24 h or 48 h. Finally, 1 mM AICAR was added after 12 h of MPP^+^ treatment. *p < 0.05, ** *p* < 0.01, *** *p* < 0.001.

To investigate whether TMEM166-induced mitophagy affects MPP^+^-induced oxidative stress, we knocked down TMEM166 in the stably expressing cell line 166-1^#^, followed by treatment with 1 mM MPP^+^ for 24 h and assessed the production of ROS. Knockdown of TMEM166 significantly reduced the production of ROS in 166-1^#^ cells (*p* < 0.001, Figure 5A,B). Next, we treated 166-1^#^ or SH-SY5Y cells with the mitophagy inhibitor Cyclosporin A (CsA) combined with MPP^+^ and assessed the production of ROS. Under this condition, CsA inhibits mitophagy by preventing mitochondrial permeability transition and inhibiting mitochondrial depolarization [28]. It was found that CsA treatment significantly increased Tom20 protein levels (*p* < 0.001, Figure 5E,F) and promoted ROS production (*p* < 0.001, Figure 5C,D) in 166-1^#^ cells and SH-SY5Y cells exposed to MPP^+^, indicating that TMEM166-promoted mitophagy is not the cause of the aggravation of MPP^+^-induced oxidative stress, instead, which clears the damaged mitochondria and protects SH-SY5Y cells from producing more ROS. In short, TMEM166-promoted mitophagy attenuates MPP^+^-induced oxidative stress.

Growing evidence proves that AMPK (adenosine 5′-monophosphate (AMP)-activated protein kinase) inhibits MPP^+^-induced oxidative stress and confers neuroprotection in SH-SY5Y cells [23,24,25,26]. We detected the activation level of AMPK during MPP^+^ treatment and found that with the increase in the MPP^+^ treatment concentration, the p-AMPK level decreased first and then gradually increased, accompanied by a upregulation first and then a gradual decrease in the TMEM166 protein level (Figure 5G,H), implying that they are functioning against each other in some way. Therefore, we overexpressed TMEM166 in SH-SY5Y cells with Myc-TMEM166 transfection and detected the activation of AMPK upon MPP^+^ treatment. As expected, the p-AMPK level was significantly reduced in the TMEM166-overexpressing group (*p* < 0.01, Figure 5I,J). We speculated that the inactivation of AMPK induced by TMEM166 overexpression possibly aggravated MPP^+^-induced oxidative stress. Using the AMPK activator AICAR to re-activate AMPK (*p* < 0.001, Figure 5I,J), we found that ROS production was significantly reduced (*p* < 0.001, Figure 5K,L), ATP production capacity and cell viabilty were significantly enhanced (Figure 5M,N) in TMEM166-overexpressing SH-SY5Y cells exposed to MPP^+^. Taken together, these results suggest that inactivation of AMPK induced by TMEM166 overexpression aggravates MPP^+^-induced oxidative stress in SH-SY5Y cells.

### 3.6. TMEM166 Inhibits AMPK Activation in Mitochondria

**Figure 6:**
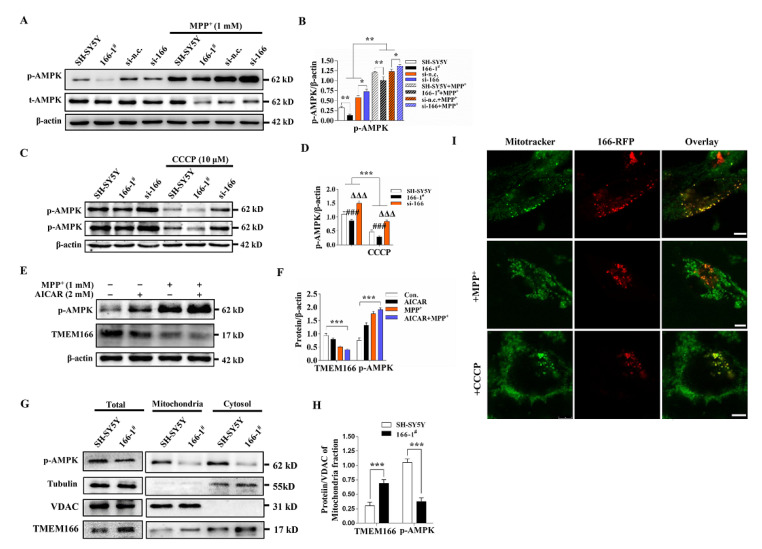
TMEM166 down-regulates AMPK activity in mitochondria. (**A**,**B**) p-AMPK levels in 166-1# cells and TMEM166 knockdown SH-SY5Y cells treated with 1 mM MPP^+^ for 24 h. * *p* < 0.05, ** *p* < 0.01. (**C**,**D**) p-AMPK levels in 166-1^#^ cells and si-166 cells treated with 10 μM CCCP for 6 h. *** *p* < 0.001, *^###^ p* < 0.001 vs. SH-SY5Y, ^ΔΔΔ^ *p* < 0.001 vs. SH-SY5Y. (**E**,**F**) p-AMPK and TMEM166 levels in SH-SY5Y cells treated with 2 mM AICAR for 5 h or 1 mM MPP^+^ for 24 h or cotreated with both. *** *p* < 0.001 (**G**) p-AMPK and TMEM166 levels in the mitochondria or cytosol of SH-SY5Y cells or 166-1^#^ cells. VDAC was used as a mitochondrial marker and Tubulin as a cytosol marker. (**H**) The qualification analysis of p-AMPK levels of mitochondria from (**C**). *** *p* < 0.001. (**I**) Observation of the colocalization of mitochondria with TMEM166 molecules. SH-SY5Y cells transfected with TMEM166-RFP plasmid (166-RFP) were treated with MPP^+^ (1 mM, 24 h) or CCCP (10 μM, 6 h) or untreated, stained with MitoTracker and we performed the live cell imaging. Scale bars, 5 μm.

To further clarify the regulation of TMEM166 on AMPK, we knocked down TMEM166 in SH-SY5Y cells and found that AMPK activation was significantly enhanced, whether being exposing to MPP^+^ or not (Figure 6A,B). Similarly, when cells were treated with the mitochondrial uncoupling agent CCCP, the phosphorylation level of AMPK in 166-1^#^ cells was still lower than that in SH-SY5Y cells and knockdown of TMEM166 also enhanced AMPK activation (Figure 6C,D). All these data indicate that TMEM166 is a negative regulator of AMPK in SH-SY5Y cells. Notably, unlike MPP^+^, CCCP treatment did not activate AMPK but inhibited its activation, probably because the rate of oxidative phosphorylation was already very high during respiratory chain uncoupling, suppressing the basal level of AMPK activation in a negative feedback manner.

We also investigated whether AMPK has a regulatory effect on TMEM166. We detected the expression of TMEM166 after activating AMPK with AICAR and found that p-AMPK levels were negatively correlated with TMEM166 protein levels. When AICAR activated AMPK, TMEM166 protein levels were significantly decreased (Figure 6E,F), which was very similar to the effect of MPP^+^ treatment on AMPK activation and TMEM166 protein. AICAR and MPP^+^ combined treatment induced synergistic effects and more TMEM166 protein was degraded (Figure 6E,F). AMPK activation with AICAR perhaps promotes autophagy and then degrades TMEM166. It could also support the high level of autophagy after TEMEM166 decreased in SH-SY5Y cells.

To find preliminary evidence for the association between TMEM166 and AMPK, we investigated their intracellular localization. AMPK can localize to mitochondria and regulate the TCA cycle directly [29]. TMEM166 was originally identified to localize in the endoplasmic reticulum (ER) [11]. Considering that overexpressed TMEM166 directly changed the morphology of mitochondria, impaired mitochondrial function and promoted mitophagy, we speculate that some of TMEM166 may localize to mitochondria and directly or indirectly suppress AMPK activation. By observing the colocalization of TMEM166-RFP and mitochondria in live SH-SY5Y cells with MPP^+^ treatment or not, we found that they had obvious colocalization in the dot structure (or in the non-vesicle structure, a linear shape of some mitochondria (green) overlaid with TMEM166 (red)) (Figure 6I and Figure S2). Under treatment with the mitophagy inducer CCCP, colocalization was also obvious (Figure 6I). These data suggest that some TMEM166 molecules per se localize to mitochondria, independent of exogenous stimuli. Furthermore, we extracted mitochondrial proteins, performed Western blotting and found that TMEM166 was, indeed, present in the mitochondrial proteins (Figure 6G,H). The most important finding is that in mitochondria, TMEM166 protein levels in 166-1^#^ cells were still significantly higher than in SH-SY5Y cells, while activated AMPK levels in 166-1^#^ cells significantly decreased (Figure 6G,H). These results indicate that TMEM166 attaches to mitochondria to suppress AMPK activation. The results also provide proof that TMEM166 may be directly involved in mitophagy.

The above data suggest that the amount of TMEM166 in mitochondria controls AMPK activation in mitochondria in SH-SY5Y cells. TMEM166 is a negative regulator for AMPK. In the absence of energy, down-regulation of TMEM166 by autophagic degradation is necessary for AMPK activation.

## 4. Discussion

**Figure 7:**
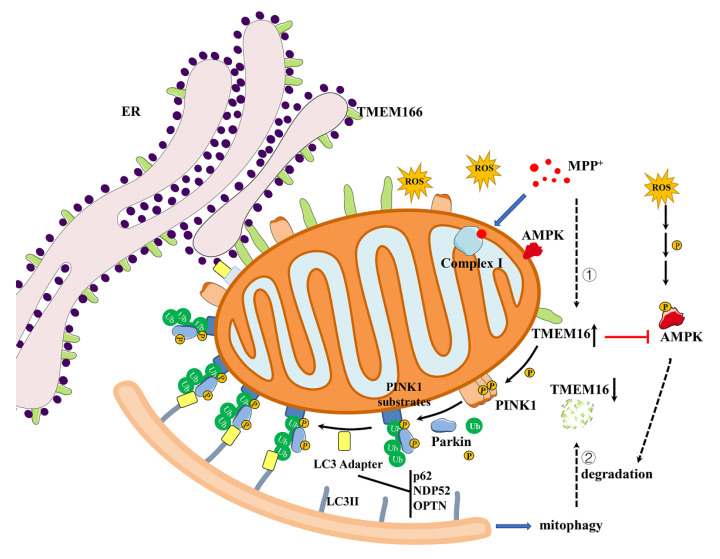
A model of TMEM166 regulation in MPP^+^-induced oxidative stress. TMEM166 mainly localizes in ER and partially in mitochondria. MPP^+^ binds to mitochondrial complex I and blocks the electron transfer of oxidative phosphorylation, leading to the production of ROS, which could eventually activate AMPK to promote cell survival. MPP^+^-induced cell oxidative stress could be divided into two stages. ① The first is the stage in which the generated ROS starts to damage the mitochondria and mitochondrial energy production decreases slightly. At this time, TMEM166 is up-regulated to promote mitophagy dependent on PINK1/Parkin to clear damaged mitochondria to maintain cell homeostasis. ② The second is that ROS has been generated enormously and the reduction in mitochondrial energy production has threatened cell survival. At this time, excessive TMEM166 in mitochondria will inhibit AMPK activation, causing mitochondrial dysfunction and oxidative stress aggravation. In this stage, more and more damaged mitochondria are produced and need to be cleared by mitophagy. The expression of TMEM166 is actually very high, but the rate of mitophagic/autophagic degradation exceeds the rate of its synthesis, causing its protein level to drop and AMPK activation to increase, which could promote mitochondrial biogenesis and produce more ATP, to maintain cell survival. In addition, activated AMPK promotes TMEM166 degradation, possibly through an AMPK-dependent autophagy/mitophagy pathway.

The present study clarifies the complex interplay between TMEM166, AMPK and mitophagy in the in vitro oxidative stress model of the parkinsonian mimetic MPP^+^. Our results reveal that the ER transmembrane protein TMEM166 can be translocated to mitochondria and negatively regulate AMPK activity. MPP^+^-treatment triggers autophagy, which degrades TMEM166 to facilitate AMPK activation, thereby exerting protection against oxidative stress. On the other hand, a genetic increase in TMEM166 protein will exacerbate oxidative stress induced by MPP^+^ by inhibiting AMPK activation, resulting in mitochondrial dysfunction and mitophagy dependent on PINK1-Parkin (Figure 7). At the beginning of MPP^+^-induced oxidative stress, TMEM166 is first up-regulated to promote mitophagy, as a priority and most basic means for maintaining cellular homeostasis. As stress intensifies, autophagy/mitophagy is further enhanced, TMEM166 is accelerated to be degraded, to activate AMPK, as a necessary and supplementary means for maintaining cell survival. This study identifies a novel function of TMEM166 in neuronal oxidative stress as an AMPK suppressor, independent of its previously identified autophagy regulation role.

Neuronal oxidative stress caused by mitochondrial dysfunction plays a vital role in the development of PD [30]. Restoring mitochondrial function and repressing oxidative stress have emerged as crucial therapeutic strategies to ameliorate neuronal damage in PD [31]. AMPK can inhibit MPP^+^-induced oxidative stress and maintain mitochondrial homeostasis [26]; it is also widely involved in mitochondrial biogenesis, mitochondrial fission and mitophagy [22]. Accumulating evidence shows that autophagy exerts neuroprotection in oxidative-stress-associated PD [32,33], where autophagy clears the damaged mitochondria, thereby preventing further deterioration in mitochondrial homeostasis [34]. Nevertheless, how AMPK is activated in oxidative-stress-associated PD and the interplay between AMPK, autophagy and oxidative stress remains unclear. According to previous studies [35,36,37,38,39], we confirmed the production of ROS and mitochondrial depolarization in MPP^+^-exposed SH-SY5Y cells and AMPK was activated by oxidative stress induced by MPP^+^ [26].

Using this cellular PD model, we revealed the two distinct roles of TMEM166 in response to mitochondrial oxidative stress, mitophagy promoter and AMPK suppressor. Under MPP^+^ treatment, although the transcript of TMEM166 was up-regulated, its protein level increased first and then decreased gradually. With lysosome inhibitor CQ treatment, the degradation of TMEM166 was significantly slowed down, indicating that TMEM166 is degraded by autophagy, which prompted us to explore its functions other than autophagy regulation. Previous studies have shown that TMEM166 transient overexpression induces apoptosis, so we constructed TMEM166 stable expression cell lines, which showed no obvious apoptosis, but prolonged S phase, mitochondrial swelling and decreased ATP production. Upon MPP^+^-treatment, the TMEM166 stably expressing cells produced more ROS, caused more severe mitochondrial depolarization and decreased cell viability. Next, we found that overexpressed TMEM166 could promote autophagy and mitophagy flux and inhibition of mitophagy promoted ROS production under MPP^+^ treatment, so mitophagy is not the cause for TMEM166-induced oxidative stress aggravation. Further, we found both knockdown TMEM166 and activation of AMPK with AICAR reduced ROS production induced by MPP^+^ and partially restored mitochondrial ATP production function and found TMEM166 could negatively regulate AMPK activation. Therefore, inactivation of AMPK by overexpressed TMEM166 resulted in the aggravation of oxidative stress induced by MPP^+^. Mitochondrial dysfunction in stably overexpressed TMEM166 cells may also be the result of long-term inhibition of AMPK activity by TMEM166. In fact, TMEM166 was partially present in mitochondria, regardless of MPP^+^ or CCCP exposure or under normal conditions and, under acute cellular energy stress, AMPK could be translocated to the mitochondria to promote energy generation [29,40]. Therefore, it is reasonable to see a decrease in activated AMPK levels in mitochondria when the amount of TMEM166 is artificially increased in mitochondria. The insufficient AMPK activation in mitochondria induced excessive ROS production under MPP^+^ treatment. How does TMEM166 inhibit AMPK activity? Directly or indirectly? The specific molecular mechanism needs to be studied in the future.

This study verified the role of TMEM166 in promoting autophagy. Unlike previous studies [11,12], we found that in addition to promoting autophagosome formation, TMEM166 is necessary for basal autophagic degradation. In addition, we found that TMEM166 can promote mitophagy, which is closely related to its localization in mitochondria. The hardest question to understand is that TMEM166 is required for autophagic degradation and is itself degraded by autophagy. This question prompts us to the autophagy adaptor molecule p62, which is also required for autophagy and degraded by autophagy along with the substrate sequestered by p62. Recent studies have shown that up-regulation of p62 promotes autophagy or promotes mitochondrial fission, facilitates mitophagy, thereby prolonging the lifespan of C. elegans and Drosophila [41,42]. Therefore, we predicted that TMEM166 might be an autophagic adaptor or mitophagic adaptor, like p62, and its degradation rate can reflect the level of autophagy or mitophagy. Further research is needed to confirm this prediction in the future.

TMEM166 was first reported to be an ER transmembrane protein that promotes apoptosis and autophagy [11]. Some studies reported that knockdown of TMEM166 leads to the impairment of mitochondrial energy production [19,20], suggesting a relationship between TMEM166 and mitochondria. This connection may be related to the communication between ER and mitochondria. Recent studies have shown that mitochondria form close physical contacts with a specialized domain of the ER, known as the mitochondria-associated membrane (MAM) [43], and this association constitutes a key signaling hub to regulate a plethora of fundamental cellular processes, such as Ca^2+^ homeostasis, lipid synthesis and mitophagy [43,44,45]. Intriguingly, many of these cellular processes are perturbed in neurodegenerative diseases and increasing evidence highlights that alterations in ER-mitochondria signaling contribute to these diseases, including PD. For instance, PINK1 and Parkin, two of the best characterized mitophagy players, accumulate at ER–mitochondria contact sites and modulate organelle crosstalk and their mutations are associated with PD [43,46]. We hypothesized that MAM might be the attachment site of TMEM166. As predicted, in our cellular model, TMEM166 partly localizes to the mitochondria, which may be involved in the formation of the MAM structure. TEMM166 promotes mitophagy by upregulating PINK1/Parkin signaling, which may be caused by alterations in TMEM166 protein levels in MAM, affecting the structure of ER–mitochondria contact and perturbing some functions of MAM. This speculation needs further investigation and verification in the future.

Given that TMEM166 is a tumor suppressor gene [14,15,16,17], we investigated the apoptosis and cell cycle in TMEM166 stable expression cell lines. Our data showed an increased distribution of cells in the S phase, indicating a special role for TMEM166 in cell division, which was consistent with a previous study where transient overexpression of TMEM166 can activate p53 and block the cell cycle [14], but stable expression of TMEM166 did not cause significant apoptosis. The discrepancies could be due to the mild overexpression of TMEM166, while the S phase arrest caused by TMEM166 may be due to TMEM166 inhibiting AMPK activation, which is required for S-phase protein synthesis and DNA replication preparation [40,47,48] and insufficient AMPK activation would make cells take longer to enter mitosis.

TMEM166 is a regulator both in autophagy and apoptosis [11] and its expression level and duration in cells determine the cells’ fate. When expressed in excess, it can also cause cell death, which is called autophagic apoptosis. Autophagic apoptosis can also be observed in animals and patients with PD [49]. From this perspective, except for apoptosis, excessive autophagy may be another threat to neurons under oxidative stress. Therefore, upon MPP^+^ treatment, SH-SY5Y cells choose to degrade TMEM166 to avoid autophagic apoptosis caused by the continuous accumulation of TMEM166 protein due to the rise of its transcripts. This explanation is consistent with the previous study that Artemisinin attenuated apoptosis by inhibiting autophagy in MPP^+^-treated SH-SY5Y cells.

## 5. Conclusions

The main finding of this study is that TMEM166, an ER transmembrane protein, can translocate to mitochondria to negatively regulate AMPK activity and induce mitochondrial dysfunction, which aggravates MPP^+^-induced oxidative stress. Neuronal cells choose to degrade TMEM166 through autophagy in response to oxidative stress to fully activate AMPK to regulate energy metabolism, thereby improving oxidative stress and promoting survival. Accumulating evidence from preclinical PD models suggests that AMPK activators have broad neuroprotective effects [50]. This study provides a basis for enhancing the therapeutic effects of AMPK-targeted drugs by reducing TMEM166 and suggests that the degradation disorder of TMEM166 may be involved in the progression of PD.

## Data Availability

Not applicable.

## Supplementary Materials

The following supporting information can be downloaded at: https://www.mdpi.com/article/10.3390/cells11172706/s1, Figure S1: Observation of LC3-mCherry puncta and autolysosome; Figure S2: TMEM166 is located in mitochondria.
