# Supplementary material for: The Degradation of TMEM166 by Autophagy Promotes AMPK Activation to Protect SH-SY5Y Cells Exposed to MPP+
Source: Cells. 2022 Aug 30;11(17):2706. doi: 10.3390/cells11172706 (PMC9454683; doi:10.3390/cells11172706)
Supplement: Supplementary file 1 [file cells-11-02706-s001.zip › Supplementary Figure and legends (1).pdf]

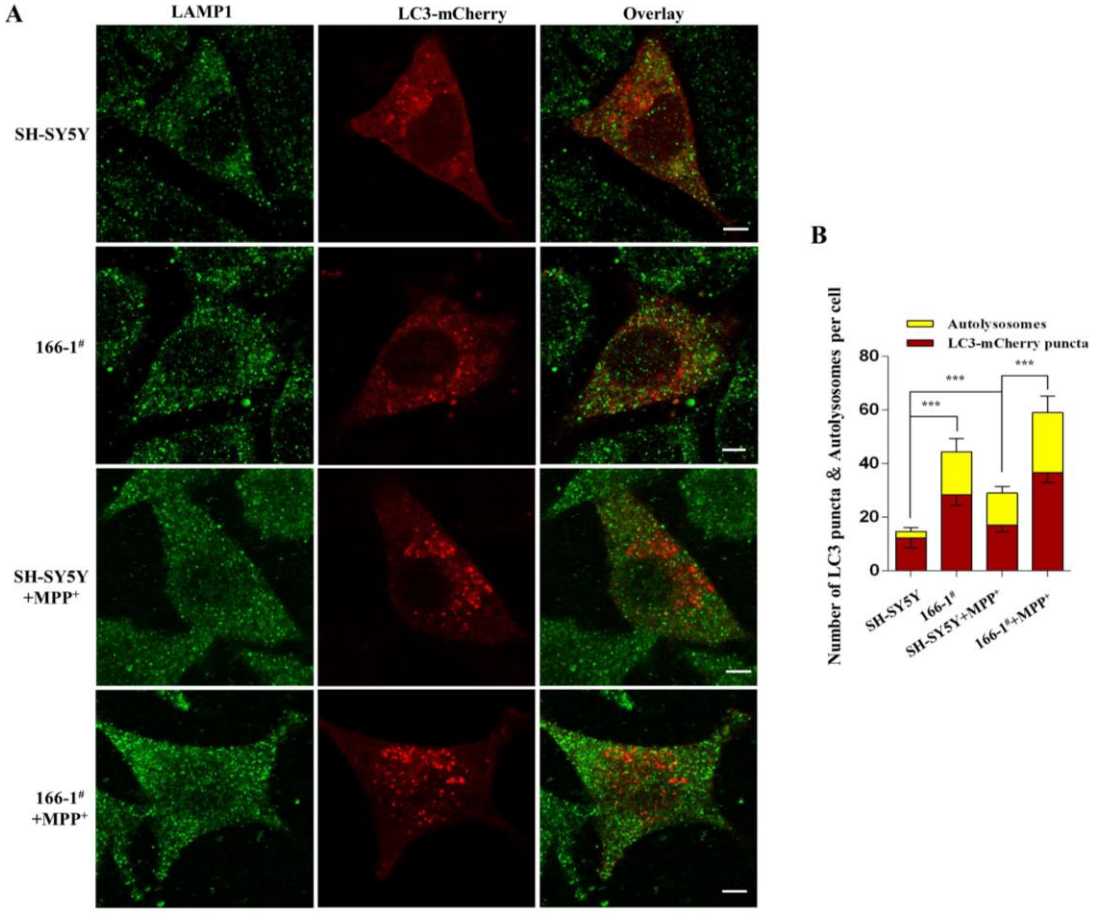

Supplementary Figure S1. Observation of LC3-mCherry puncta and autolysosome. (A) SH-SY5Y cells and 166-1# cells transfected with LC3-mCherry plasmids were untreated or treated with 1 mM MPP^+ for 24 h, then fixed and stained with LAMP1 antibody, and were imaged by confocal microscopy. Scale bars, 5 μm. (B) Statistical analysis of the number of LC3-mCherry dots and the colocalized dots of LC3-mCherry with LAMP1 per cell. *** $p<0.001$

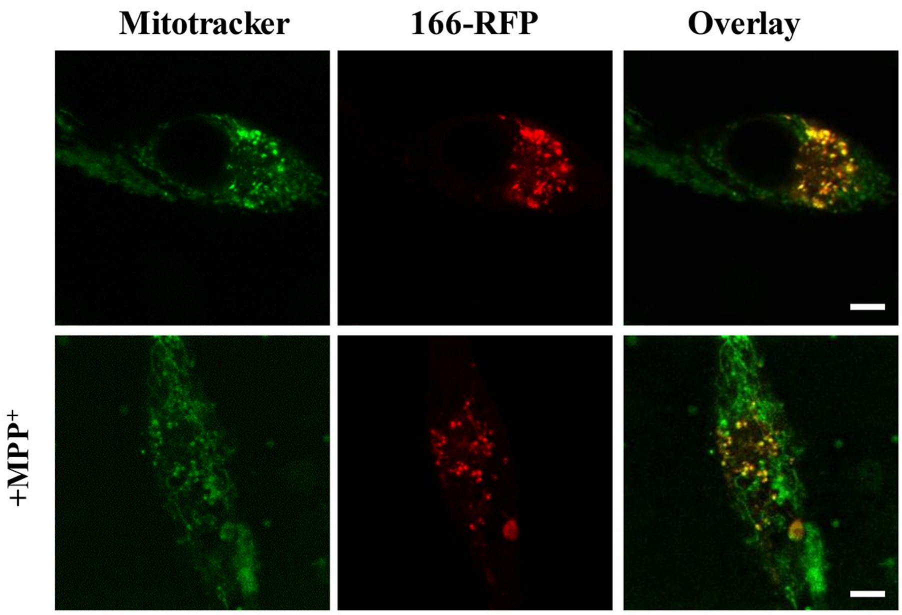

Supplementary Figure S2. TMEM166 is located in mitochondria. Observation of the co-localization of mitochondria and TMEM166 molecules in SH-SY5Y cells transfected TMEM166-RFP plasmid with or without MPP^+ treatment (1 mM, 24 h). Note that TMEM166 appears to have linear shapes similar to that of mitochondria. Scale bars, 5 µm.
